# Neonatal Selenoenzyme Expression Is Variably Susceptible to Duration of Maternal Selenium Deficiency

**DOI:** 10.3390/antiox10020288

**Published:** 2021-02-14

**Authors:** Laura G. Sherlock, Durganili Balasubramaniyan, Lijun Zheng, Miguel Zarate, Thomas Sizemore, Cassidy Delaney, Trent E. Tipple, Clyde J. Wright, Eva Nozik-Grayck

**Affiliations:** 1 Perinatal Research Center, Department of Pediatrics, University of Colorado Anschutz Medical Campus, Aurora, CO 80045, USA; durganili.balasubramaniyan@cuanschutz.edu (D.B.); lijun.zheng@cuanschutz.edu (L.Z.); miguel.zarate@cuanschutz.edu (M.Z.); Thomas.sizemore@colorado.edu (T.S.); clyde.wright@cuanschutz.edu (C.J.W.); 2 Cardiovascular Pulmonary Research Laboratories, Departments of Pediatrics and Medicine, University of Colorado Anschutz Medical Campus, Aurora, CO 80045, USA; cassidy.delaney@cuanschutz.edu; 3 Department of Pediatrics, University of Oklahoma College of Medicine, Oklahoma City, OK 73104, USA; trent-tipple@ouhsc.edu (T.E.T.); eva.nozik@cuanschutz.edu (E.N.-G.)

**Keywords:** selenium, antenatal nutrition, periconception nutrition, glutathione peroxidase, thioredoxin reductase, selenocysteine processing, liver, lung, redox state

## Abstract

Maternal selenium (Se) deficiency is associated with decreased neonatal Se levels, which increases the risk for neonatal morbidities. There is a hierarchy to selenoprotein expression after Se deficiency in adult rodents, depending on the particular protein and organ evaluated. However, it is unknown how limited Se supply during pregnancy impacts neonatal selenoprotein expression. We used an Se-deficient diet to induce perinatal Se deficiency (SeD), initiated 2–4 weeks before onset of breeding and continuing through gestation. Neonatal plasma, liver, heart, kidney, and lung were collected on the day of birth and assessed for selenoproteins, factors required for Se processing, and non-Se containing antioxidant enzymes (AOE). Maternal SeD reduced neonatal circulating and hepatic glutathione peroxidase (GPx) activity, as well as hepatic expression of Gpx1 and selenophosphate synthetase 2 (Sps2). In contrast, the impact of maternal SeD on hepatic thioredoxin reductase 1, hepatic non-Se containing AOEs, as well as cardiac, renal, and pulmonary GPx activity, varied based on duration of maternal exposure to SeD diet. We conclude that the neonatal liver and circulation demonstrate earlier depletion in selenoenzyme activity after maternal SeD. Our data indicate that prolonged maternal SeD may escalate risk to the neonate by progressively diminishing Se-containing AOE across multiple organs.

## 1. Introduction

The in-utero environment impacts the short- and long-term health of the fetus and neonate. Malnutrition before and during pregnancy regulates the supply of macro- and micronutrients to the fetus, which can result in poor outcomes for the infant [1,2,3,4]. Clinical studies have linked deficiency of the micronutrient selenium (Se) to multiple neonatal morbidities, including sepsis, impaired neurodevelopment, poor postnatal growth, bronchopulmonary dysplasia, and retinopathy of prematurity [5,6,7,8,9,10,11]. Se is a trace mineral essential for redox homeostasis and response to oxidative stress [12,13]. An impaired ability to respond to oxidative challenges is associated with morbidity and mortality in the neonatal period, and it is possible that decreased Se-associated antioxidant enzymes (AOE) may contribute to disrupted organogenesis and injury during infancy [14,15,16,17]. However, Se deficiency can occur concordantly with poor overall nutrition [18]. Thus, clinical models are limited in their ability to dissect the specific effect of Se, necessitating preclinical models that can isolate the direct impact of Se deficiency on neonatal morbidities.

The regulation of Se processing has been well characterized in pre-clinical adult rodent models. Se intake occurs through dietary consumption of food or supplements, in the forms of selenomethionine, sodium selenite, or sodium selenate [19]. After absorption through the gut, Se is processed in the liver, then transported to organs through circulating selenoprotein P (Selenop), a Se enriched protein consisting of 10 Se moieties [20]. Se processing in the liver requires a multiple step process requiring cis- and trans- factors to facilitate the addition of Se into a selenocysteine (Sec) specific tRNA before incorporation into Se-containing proteins [19,21,22]. The incorporation of Se into Se-containing AOE confers their enzymatic activity. Several of the selenoenzymes are known to decrease after limited Se supply in adults [13,23,24].

There is a specific hierarchy of selenoprotein expression after Se deficiency in adult rodents, where certain Se-containing proteins are quickly diminished, while others are preserved [25]. The relationship between Se and selenoprotein expression varies depending on the target tissue, organ, and sex [13,25,26]. Depending on the organ evaluated, the decrease in selenoenzymes can lead to increased oxidative stress as well as dysfunction in the response to injury [27,28,29,30,31,32,33]. The liver is one of the most Se enriched organs and Se is quickly depleted after dietary deficiency [34,35]. In the adult liver, Se deficiency induces transcriptionally regulated decreases in the antioxidant enzymes glutathione peroxidase 1 (GPx1) and thioredoxin reductase 1 (Txnrd1) [35,36,37]. Additionally, in response to Se deficiency, the adult liver exhibits a concomitant increase in the activity of non-Se containing AOEs, including superoxide dismutase (SOD) and heme-oxygenase 1 (HO-1) [24,38].

Maternal Se status regulates Se levels in the neonate, and maternal circulating Selenop is responsible for placental Se transport [18,39,40,41,42]. Understanding of specific neonatal selenoproteins expression in response to maternal Se deficiency may provide insight into mechanisms by which Se deficiency is associated with worse outcomes in the neonatal period. We sought to determine how the neonate responds to limited maternal Se supply, including the expression of proteins required for the processing of Se, the hierarchy of organ-specific selenoproteins, as well non-Se associated AOE. We hypothesized that the neonatal liver responds to maternal Se deficiency by decreasing hepatic expression of GPx1 and Txnrd1 expression, and by increasing expression of non-Se containing AOE, similar to the adult.

## 2. Materials and Methods

### 2.1. Model of Perinatal Se Deficiency

C57Bl/6 female and male mice consumed standard rodent chow during gestation and lactation. They were weaned between 3 and 4 weeks of life and allocated to either a Torula yeast based Se sufficient (SeS) diet that provides 0.4 ppm sodium selenite or an Se-deficient (SeD) diet, with <0.01 ppm sodium selenite. Simple randomization was used to determine if a mouse received SeS or SeD diets. These mice were fed SeS or SeD diets for 2–4 weeks, then livers and plasma were assessed at 6–8 weeks of age to confirm Se deficiency at the time breeding would be set in future experiments. Subsequent studies also allocated 3–4-week-old female and male mice to the SeS (0.4 ppm sodium selenite) or SeD (<0.01 ppm sodium selenite) diets for 2–4 weeks before breeding was set. They were allowed to naturally deliver, and pups were assessed on day of birth. A single male and single female mouse were randomly selected and paired for breeding. Vaginal plugs were not evaluated to determine precise information regarding days of pregnancy, however the majority of SeS and SeD breeding dams became pregnant in the first month after being paired. Breeding pairs were allowed to sequentially gestate. Pups were sexed visually on the day of birth and sex was confirmed for pups used in experiments by measuring *Sry* mRNA. Sry mRNA was measured in hepatic tissue. If the cycle threshold was ≤35, mice were confirmed to be male. Mice from 10 sufficient litters and 10 deficient litters were assessed. A total of 20 SeS neonatal pups and 32 SeD pups were assessed. To test for the impact of the antenatal environment, results for two randomly selected female pups or two male pups from each litter were averaged.

### 2.2. Ethical Considerations of Animal Care

All procedures were approved by the Institutional Animal Care and Use Committee (IACUC, protocol number 956) at the University of Colorado (Aurora, CO, USA). The National Institutes of Health guidelines for ethical animal treatment was followed in regard to the care and handling of the animals [43].

### 2.3. Collection of Blood and Organs

Animals were sacrificed using an intraperitoneal (IP) dose of pentobarbital sodium (200 mg/kg). To collect blood, the right ventricle of the heart was punctured through an open chest, blood collected in heparinized tubes, and placed on ice. These samples were then spun at 2000 rpm × 10 min at 4 °C After collecting blood, perfusion of the pulmonary artery was achieved with 5 mL phosphate buffered saline (PBS), flushing the lungs. Lungs were visually confirmed to be white before collection. Organs were placed in collection tubes and snap frozen. Plasma and organs were stored at −80 degrees Celsius for less than 9 months prior to analysis.

### 2.4. Glutathione Peroxidase Activity

Glutathione peroxidase activity was measured in the plasma, liver, heart, kidney, and lung as previously described [44]. GPx activity was measured indirectly by measuring the decrease in absorption for NADPH at 340 nm, in a coupled reaction with glutathione reductase. Briefly, known quantities of reduced glutathione, glutathione reductase, and NADPH were added in reaction buffer. 222.5 μL of this reaction buffer was added to 17.5 μL of sample. Sample plus reaction buffer was then incubated for 10 min at 37 °C. Hydrogen peroxide was used as a catalyst, at a final concentration of 50 μM. As the NADPH in the reaction buffer is oxidized to NADP+, a reduction in the absorbance at 340 nm occurs. The reaction was monitored for 2 min on a microplate reader heated to 37 °C. Based on the difference in absorption at 340 nm, GPx activity was calculated using Lambert–Beer’s law. For this, 1 U of GPx activity was defined as the consumption of 1 μmol NADPH/min/mL.

For sample preparation, neonatal plasma was diluted 1:2 with deionized water. Ten to 20 mg of liver, heart, kidney, or lung was lysed in 200 μL Tris/Hcl 50 mM with 5 mM EDTA and 1 mM sodium azide, as well as Halt protease and phosphatase inhibitors (ThermoFisher, Waltham, MA, USA) (1:100). Lysates were spun at 14,000 G for 10 min, supernatant was removed, and protein content was determined by Pierce BSA assessment (ThermoFisher, Waltham, MA, USA). Samples were diluted to a concentration of 2 μg /μL. Then, 17.5 μL of this solution, or 35 μg of protein, was used for the assay.

### 2.5. Immunoblot Analysis

After protein isolation from the liver, between 30 to 50 micrograms of protein were used for analysis. This protein was electrophoresed on a 4–12% polyacrylamide gel (Invitrogen, Waltham, MA, USA) and then transferred on to an Immobilon membrane (Millipore, Burlington, MA, USA). To evaluate the total amount of protein transferred, Revert Total Protein Stain (LiCor, Lincoln, NE, USA) was used. This determination of total protein was used to normalized the measured proteins and all images of primary antibodies as well as total protein stains are available in the supplemental file. Blots were then exposed to primary antibodies at 4 °C overnight or over the weekend. Information regarding the antibody and dilution are available in Supplementary Table S1. Secondary antibodies were in the appropriate host (1:5000). Imaging was preformed using the LiCor Odyssey system (Licor, Lincoln, NE, USA). Densitometric analysis was performed using ImageStudio (LiCor, Lincoln, NE, USA).

### 2.6. Analysis of Relative mRNA Levels by RT-qPCR

Organs that had been flash frozen and stored at −80 degrees Celsius were removed and quickly placed in RNA Lysis buffer (RLT) buffer (Qiagen). These samples were homogenized using the Bullet Blender (NextAdvance, Troy, NY, USA). The mRNA levels of whole liver were isolated using RNeasy Mini Kit (Qiagen, Germantown, MD, USA). The quality and concentration of RNA levels were evaluated using Nanodrop (ThermoFisher Scientific, Waltham, MA, USA). Complementary DNA was generated at 1 μg/20 μL with a Verso cDNA synthesis kit (ThermoFisher Scientific, Waltham, MA, USA). Quantitative real-time PCR was utilized for the evaluation of mRNA levels. This was done using exon spanning primers, Taqman gene technology, and StepOnePlus Real-Time PCR (Applied Biosystems, Foster City, CA, USA) (See Supplementary Table S1 for primers as well as temperatures and number of cycles). Quantification was performed using the cycle threshold method (DDCT), normalizing to the housekeeping gene 18S. 18S variability was low and did not differ between dietary groups.

### 2.7. Thiobarbituric Acid Reactive Substance Assay

To measure byproducts of lipid peroxidation, a thiobarbituric acid reactive substance assay was used per manufactorer’s instructions (Cayman Chemicals, Ann Arbor, MI, USA). Briefly, 20–30 μg of hepatic tissue was homogenized in radioimmunoprecipitation assay (RIPA) buffer plus protease inhibitor. The lysed samples were centrifuged for 10 min at 1600 G. Supernatant was removed and 100 μL was added to a reaction tube. 100 μL of sodium dodecyl sulfate (SDS) solution and 4 mL of color reagent was then added to the reaction tube. This was then heated to 90–100 °C for 1 h, during which thiobarbituric acid- malondialdehyde adducts form. The vials were removed and placed in an ice bath for 60 min, then centrifuged for 10 min at 1600 G. Supernatant was removed and 150 μL loaded to a 96-well plate, after which absorbance was measured at 530–540 nm.

### 2.8. Statistical Analysis

For breeding males and females, comparisons were made between sex and dietary exposure groups. The null hypothesis that no difference existed between and within groups was tested by two-way ANOVA with Tukey’s correction for multiple comparisons. For experiments on neonatal mice, comparisons were made by Student’s two-sided *t*-tests for experiments comparing two groups. When comparing three groups, comparisons were made by one-way ANOVA with Tukey’s method for multiple comparisons. When comparing more than three groups, two-way ANOVA was used. Figures are presented as mean ±SEM. Additionally figures with the 95% confidence interval are available in the supplemental file (Supplementary Figures S6–S10). For statistical significance between groups was defined at *p* < 0.05 and were analyzed using Prism (Graphpad Software, San Diego, CA, USA).

## 3. Results

### 3.1. Adult Breeding Age Mice Exposed to Se Deficient Diet Demonstrate Decrease in Circulating and Hepatic Selenoproteins

**Figure 1:**
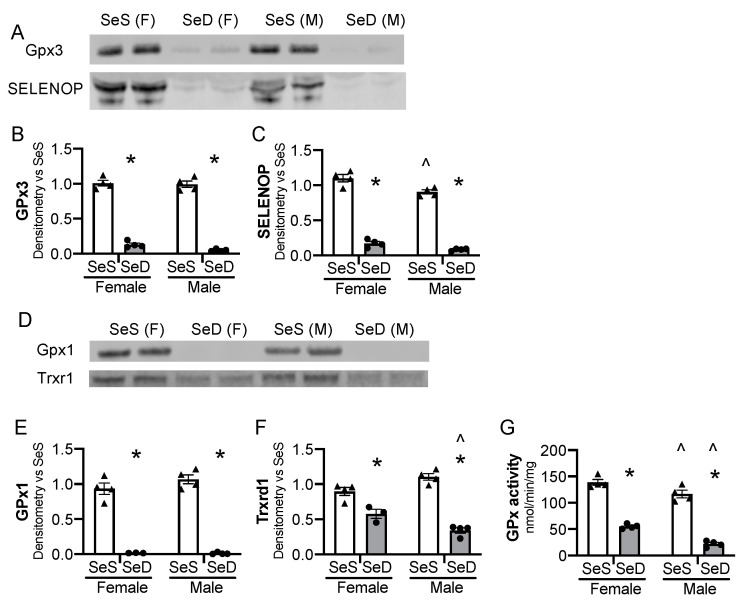
Breeding pairs on a Se deficient diet exhibit diminished selenoenzyme protein content and activity. C57Bl/6 mice were placed on diets that differed only in Se content, either 0.4 ppm or <0.01 ppm of sodium selenite for 2–4 weeks. Female and male breeders were assessed for Se status. (**A**) Representative blots of plasma GPx3 and SELENOP, assessed in 2 μL plasma. Densitometry results are normalized to total protein stain and expressed as a ratio to SeS control. Densitometric analysis of (**B**) GPx3, (**C**) SELENOP. Hepatic whole organ homogenate was evaluated. (**D**) Representative blots of hepatic Trxrd1 and Gpx1. Densitometry results are normalized to total protein stain and expressed as a ratio to SeS control. Densitometric analysis of (**E**) GPx1 and (**F**) Trxrd1 (**G**) Glutathione peroxidase activity level by oxidation of NADPH per minute per mg of protein. N = 4 for all groups. Data are presented as mean (±SEM), * *p* < 0.05 vs. sex-matched SeS control by multiple comparisons after two-way ANOVA, ∧ *p* < 0.05 vs. diet-matched F by multiple comparisons after two-way ANOVA.

We first sought to establish that mice exposed to SeD diets were deficient in selenoproteins before the onset of breeding. Female and male mice were allocated SeS or SeD diets for 2–4 weeks and were assessed at 6–8 weeks of life, to reflect the age breeding would be initiated in subsequent experiments. Gpx3 content decreased in both male and female SeD mice (Figure 1A,B). Plasma Selenop content also decreased in male and female SeD mice (Figure 1A,C). We next evaluated the hepatic protein content for the selenoenzymes glutathione peroxidase 1 (GPx1) and thioredoxin reductase 1 (Txnrd1). Hepatic GPx1 protein content decreased in both male and female SeD breeding aged mice (Figure 1D,E). Hepatic Txnrd1 also decreased after SeD, with male SeD mice demonstrating less Txnrd1 protein content than female SeD mice (Figure 1D,F). GPx was then measured. Female SeS mice demonstrated higher hepatic GPx activity than male SeS mice (Figure 1G). GPx activity dramatically decreased in the liver of both male and female SeD mice, and SeD males exhibited lower GPx activity than SeD females (Figure 1G).

### 3.2. Antenatal Se Deficiency Decreases Litter Size and Circulating GPx Activity in Neonatal Mice

**Figure 2:**
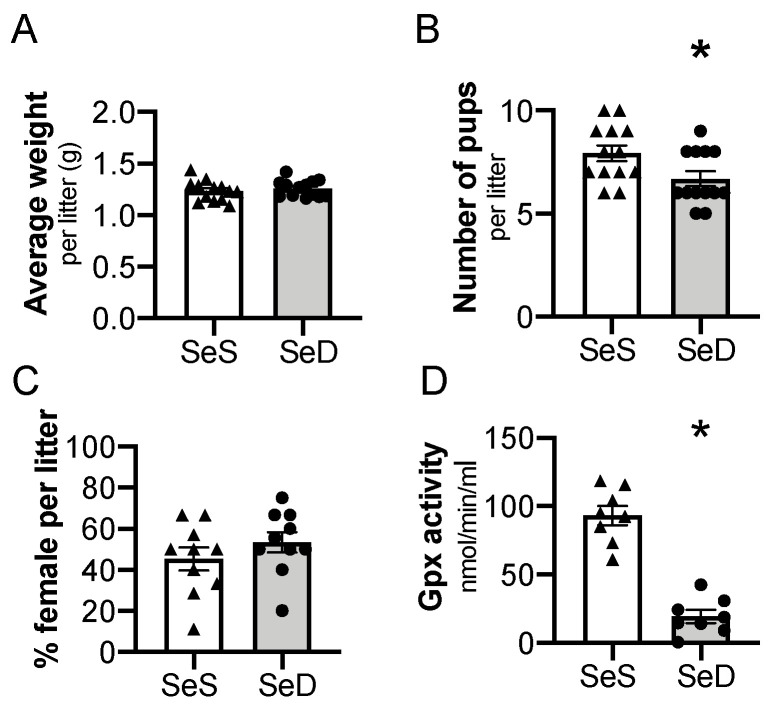
Maternal selenium (Se) deficiency is associated with decreased litter size but similar weight after birth. C57Bl/6 mice were placed on diets that differed only in Se content, either 0.4 ppm or <0.01 ppm of sodium selenite. Breeding was initiated after 2–4 weeks on diets and natural delivery was allowed. (**A**) Neonatal pup weight per litter on day of birth, (**B**) number of pups per litter on day of birth, (**C**) ratio of females per litter, (**D**) plasma glutathione peroxidase activity level by oxidation of NADPH per minute. Each data point represents either a female or male from each litter; each individual point is an average of two mice. Triangles are SeS data points and circles are SeD data points. *N* = 4–13 litters for all groups. Data are presented as mean (±SEM), * *p* < 0.05 vs. SeS control, by two-sided *t*-test.

Previous studies reported that antenatal Se deficiency decreases maternal weight gain and is associated with altered placental function [45]. To extend these observations and to test the impact of Se deficiency on neonatal pups, we evaluated the characteristics of the resulting offspring on the day of birth. There was not a difference in total body weight per litter between postnatal day 0 (P0) pups exposed to SeS or SeD antenatal diets (Figure 2A). The number of pups per litter, or litter size, of SeD pregnancies was lower than SeS pregnancies (Figure 2B). The percentage of female to male mice was not different between SeS and SeD pregnancies (Figure 2C). The neonatal pups appeared phenotypically normal. To assess Se status, plasma GPx activity was measured in pups born to SeS and SeD dams. Plasma GPx activity was significantly decreased in SeD pups (Figure 2D). There were no sex-dependent differences in GPx activity between either SeS or SeD pups (Supplementary Figure S1).

### 3.3. Neonatal Hepatic Factors for Se Processing and Transport Are Decreased after Antenatal Se Deficiency

**Figure 3:**
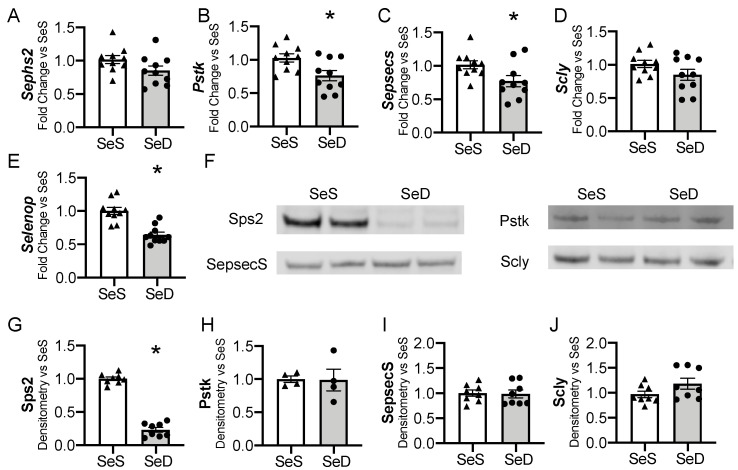
Hepatic factors for selenocysteine synthesis and Se transport in neonates born after antenatal Se deficiency. C57Bl/6 mice were placed on diets that differed only in Se content, either 0.4 ppm or <0.01 ppm of sodium selenite. Breeding was initiated after 2–4 weeks on diet and natural delivery was allowed. Hepatic organ homogenate was evaluated on day of birth. Each data point represents either a female or male from each litter; each individual point is an average of two mice. Fold change in (**A**) *Sephs2*, (**B**) *Pstk,* (**C**) *Sepsecs* mRNA, (**D**) *Scly* mRNA, and (**E**) *Selenop* mRNA are shown normalized to SeS samples. (**F**) Representative Western blots of hepatic Sps2, Pstk, SepsecS, and Scly. Densitometric analysis of (**G**) Sephs2, (**H**) Pstk, (**I**) SepsecS, and (**J**) Scly protein content expression. Results are normalized to total protein stain and expressed as a ratio to SeS mice. *N* = 4–10 for all groups. Data and presented as mean (±SEM), * *p* < 0.05 vs. SeS control, by two-sided *t*-test.

The liver is one of the first organs to demonstrate selenoprotein depletion in adult rodents exposed to Se deficiency [35,46,47]. For the liver to convert dietary Se into functioning selenoproteins, a machinery of cis- and trans- binding proteins are required to co-translationally load the Se molecule into a selenocysteine (Sec) specific tRNA and to facilitate insertion into newly translated proteins [19]. We evaluated if the hepatic machinery for Sec synthesis, recycling, or transport were altered in response to antenatal Se deficiency. Selenophosphate synthetase 2 (*Sephs2*) mRNA levels were not significantly reduced by maternal Se deficiency (*p* = 0.09), whereas phosphoseryl-tRNA kinase (*Pstk*) and selenocysteine synthase (*Sepsecs*) mRNA decreased (Figure 3A–C). The mRNA levels of selenocysteine lyase (Scly), an enzyme that recycles Se from selenoproteins, were unchanged by antenatal Se deficiency, *p* = 0.11 (Figure 3D). We measured the transcription of selenoprotein P (SelenoP), a Se rich protein that is responsible for Se transport and distribution to other tissues. *SelenoP* mRNA levels were decreased in newborn mice born to SeD dams (Figure 3E). Protein content for the hepatic machinery for Sec processing and recycling was next assessed. Expression of selenophosphate synthetase (Sps2) was significantly decreased in response to maternal Se deficiency (Figure 3F,G). Despite the decrease in mRNA, Pstk and SepsecS protein levels were unaltered by maternal Se deficiency (Figure 3F,H,I). Scly content was also unchanged after Se deficiency (Figure 3F,J). Sex differences were not observed in the baseline abundance of mRNA or protein content for the hepatic Sec machinery in SeS pups, nor in the neonatal response to maternal Se deficiency (Supplementary Figure S2).

### 3.4. Neonatal Hepatic Glutathione Peroxidase 1 (Gpx1) Is Decreased after Antenatal Se Deficiency

**Figure 4:**
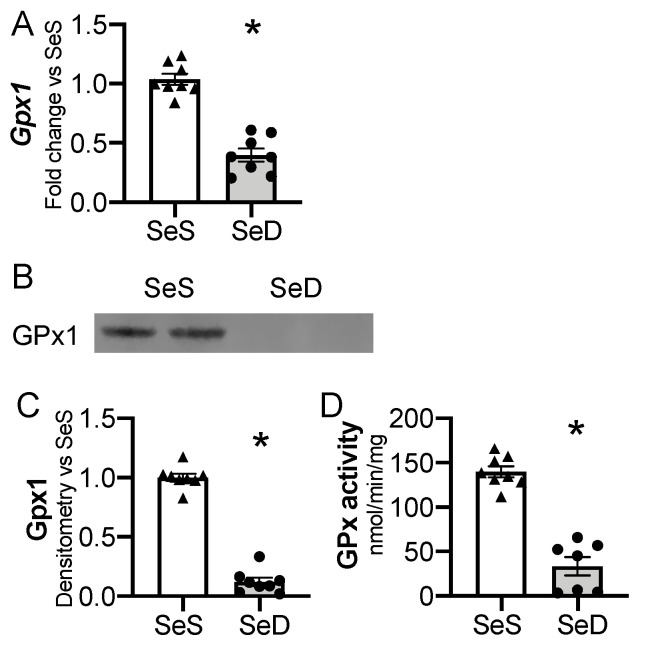
Neonatal hepatic glutathione peroxidase 1 is decreased by antenatal Se deficiency. C57Bl/6 mice were placed on diets that differed only in Se content, either 0.4 ppm or <0.01 ppm of sodium selenite. Breeding was initiated after 2–4 weeks on diet and natural delivery was allowed. Hepatic organ homogenate was evaluated on day of birth. Each data point represents either a female or male from each litter; each individual point is an average of two mice. (**A**) Fold change in Gpx1 mRNA expression is shown normalized to SeS samples. (**B**) Representative Western blot of hepatic Gpx1, Densitometric analysis of (**C**) Gpx1 protein content expression. Results are normalized to total protein stain and expressed as a ratio to Se sufficient mice. (**D**) Glutathione peroxide activity level by oxidation of NADPH per minute per milligram of protein. *N* = 7–8 for all groups. Data and presented as mean (±SEM), * *p* < 0.05 vs. SeS control, by two-sided *t*-test.

Gpx1 is highly sensitive to availability of Se supply in adult and in vitro models [24,46]. Thus, we interrogated the impact of antenatal Se deficiency on neonatal hepatic Gpx1 transcription, expression, and activity. Antenatal Se deficiency resulted in a significant decrease in hepatic *Gpx1* mRNA levels (Figure 4A), concomitant with a decrease in hepatic Gpx1 protein content (Figure 4B,C) and GPx activity level (Figure 4D). We evaluated the impact of sex on Gpx1 expression in P0 pups exposed born to SeS or SeD dams [47,48]. There were no differences in *Gpx1* mRNA, protein, or activity between female and male SeS or SeD pups (Supplementary Figure S3).

### 3.5. Prolonged Duration of Maternal Se Deficiency Decreases Neonatal Hepatic Thioredoxin Reductase 1

**Figure 5:**
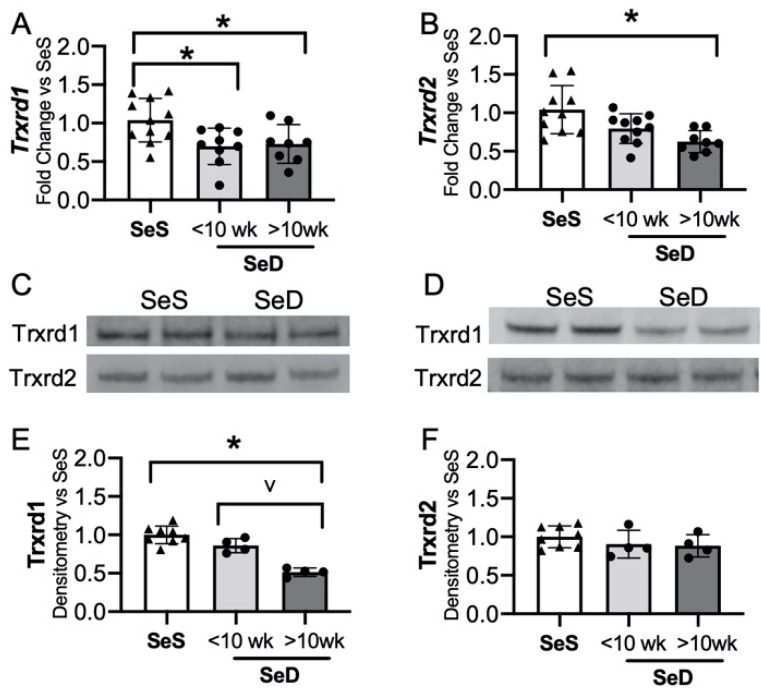
Neonatal hepatic thioredoxin reductase 1 is decreased by prolonged antenatal Se deficiency. C57Bl/6 mice were placed on diets that differed only in Se content, either 0.4 ppm or <0.01 ppm of sodium selenite. Breeding was initiated after 2–4 weeks on diet and natural delivery was allowed. Hepatic organ homogenate was evaluated on day of birth. Analysis is done with SeD samples separated based on if the breeding dam received more or less than 10 weeks SeD diet at time of birth. Each data point represents either a female or male from each litter; each individual point is an average of two mice. Fold change in (**A**) *Trxrd1* mRNA and (**B**) *Trxrd2* mRNA is shown normalized to SeS samples, (**C**) representative Western blot of hepatic Trxrd1 and Trxrd2 for SeS samples and SeD samples born to dams who received <10 weeks SeD diet, (**D**) representative Western blot of hepatic Trxrd1 and Trxrd2 for SeS samples and SeD samples born to dams who received >10 weeks SeD diet, densitometric analysis of (**E**) Trxrd1 and (**F**) Trxrd2 protein content expression. Results are normalized to total protein stain and expressed as a ratio to Se sufficient mice. N = 4–10 for all groups. Data are presented as mean (±SEM), * *p* < 0.05 vs. SeS control by multiple comparisons after one-way ANOVA, ∨ *p* < 0.05 vs. SeD <10 weeks by multiple comparisons after one-way ANOVA.

The Txnrds are selenoproteins that reduce thioredoxin and contribute to the reduction of protein disulfide bonds [19]. The transcription and activity of hepatic Txnrd1 is decreased in adult Se deficiency [24,46]. To determine if the neonatal liver was similarly impacted by low Se supply, Txnrd1 and Txnrd2 transcripts and protein contents were assessed. The initial analysis of neonatal Trxrd1 protein content after maternal SeD revealed variability in response, which appeared to relate to duration of maternal Se deficiency. As our model allowed serial breeding, with each subsequent pregnancy, the dams had a longer cumulative exposure to the dietary group. Thus, we evaluated pups in three groups: (1) Pups born to dams receiving SeS diets; (2) pups born to dams exposed to a *short* total SeD duration (<10 weeks, reflecting a dam’s first or second gestation); and (3) pups born to dams exposed to *prolonged* SeD duration (>10 weeks, reflecting a dam’s third or fourth gestation). Hepatic *Txnrd1* mRNA decreased with both short and prolonged maternal SeD exposure, whereas *Txnrd2* was decreased only following prolonged SeD exposure. (Figure 5A,B). Next, Txnrd1 and Txnrd2 protein content was measured. Pups born to dams exposed to short SeD had unchanged Txnrd1 content (Figure 5C,E). In contrast, pups born to dams exposed to a prolonged SeD duration demonstrated decreased Txnrd1 content (Figure 5D,E). Txnrd2 content was not altered by short or prolonged maternal SeD duration (Figure 5C,D,F). There was no impact of sex on Txnrd1 and Txnrd2 mRNA or protein contents in SeS or SeD pups (Supplementary Figure S4). Supplemental Figure S4G also includes the number of weeks the SeD dams were exposed to SeD diet in the short and prolonged exposure groups. These results indicate that in contrast to the adult Se deficient liver, neonatal Txnrd1 content is initially sustained following exposure to maternal Se deficiency, but eventually decreases in pups born to dams exposed to a longer duration of SeD.

### 3.6. Prolonged Maternal Se Deficiency Was Associated with Increased Expression of Superoxide Dismutase 2 and Heme Oxygenase 1

**Figure 6:**
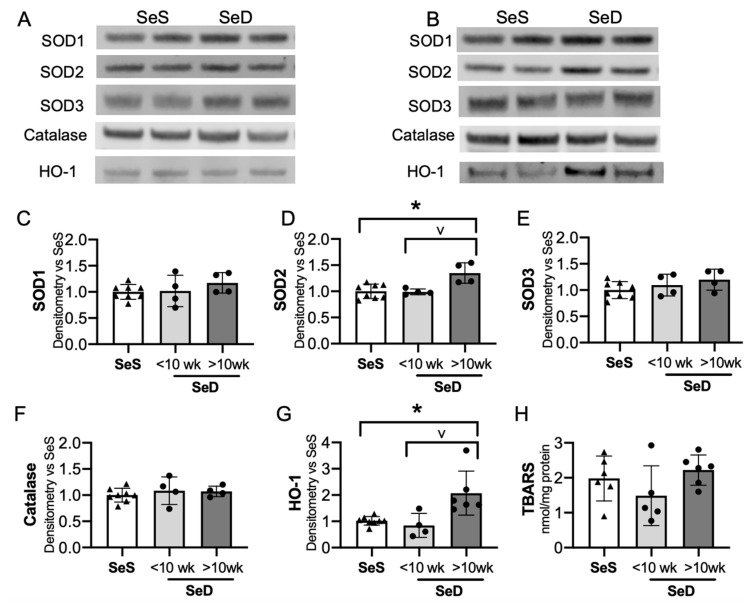
Neonates born to dams with prolonged Se deficient exhibit increased hepatic SOD2 and HO-1. C57Bl/6 mice were placed on diets that differed only in Se content, either 0.4 ppm or <0.01 ppm of sodium selenite. Breeding was initiated after 2–4 weeks on diet and natural delivery was allowed. Hepatic organ homogenate was evaluated on day of birth. Each data point represents either a female or male from each litter; each individual point is an average of two mice. (**A**) Representative Western blots of hepatic SOD1, SOD2, SOD3, catalase, and HO-1 for SeS samples and SeD samples born to dams who received <10 weeks SeD diet, (**B**) representative Western blots of hepatic SOD1, SOD2, SOD3, catalase, and HO-1 for SeS samples and SeD samples born to dams who received >10 weeks SeD diet, densitometric analysis of (**C**) SOD1, (**D**) SOD2, (**E**) SOD3, and (**F**) Catalase, (**G**) HO-1 protein content expression. Results are normalized to total protein stain and expressed as a ratio to SeS mice. (**H**) TBARS expression, expressed as a ratio to SeS mice N = 4–8 for all groups. Data are presented as mean (±SEM), * *p* < 0.05 vs. SeS control, by multiple comparison after one-way ANOVA, ∨ *p* < 0.05 vs. SeD <10 weeks by multiple comparisons after one-way ANOVA.

The adult Se deficient liver demonstrates an increase in non-Se containing antioxidant enzymes, including superoxide dismutase (SOD) activity and heme-oxygenase-1 (HO-1) protein level and activity level [24,38,49]. To evaluate if the neonatal Se deficient liver exhibited a similar response, we tested the expression of several non-Se containing antioxidant enzymes important for hepatic redox state. The hepatic expression of SOD 1 and 3 were following short or prolonged maternal exposure to Se deficiency were no different than in pups born to SeS dams (Figure 6A–C,E). In contrast, SOD2 levels were greater in pups born to dams with prolonged SeD when compared to pups born to either SeS or short SeD exposure (Figure 6A,B,D). Catalase was unaltered after short and prolonged maternal exposure to Se deficiency (Figure 6A,B,F). Finally, we evaluated HO-1 expression and found no difference in HO-1 expression after short maternal Se deficiency, but a significant increase after prolonged maternal Se deficiency. (Figure 6A,B,G). Decreased Se status and Gpx activity is associated with increased markers of oxidative stress, specifically in markers of lipid peroxidation, in several models [29,50,51]. Thus, we evaluated the effect of antenatal Se deficiency on byproducts of lipid peroxidation in the livers of pups by measuring the amount of thiobarbituric acid reactive substances (TBARS). The amount of TBARS in livers of pups born to short or prolonged SeD dams was no different than in SeS controls (Figure 6H). Finally, we did not identify sex-dependent effects on baseline protein content or abundance following Se deficiency for any of the non-Se containing antioxidants (Supplementary Figure S5). 

### 3.7. Neonatal GPx Activity in the Heart, Kidney, and Lung Is Variably Decreased Depending on Duration of Antenatal Se Deficiency

**Figure 7:**
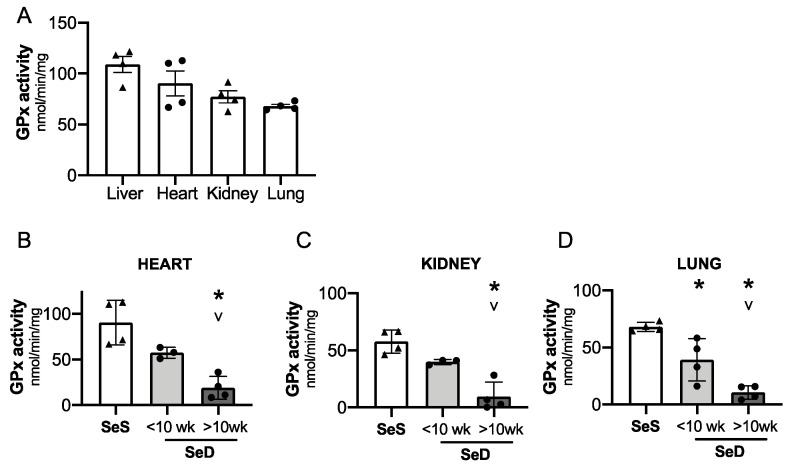
Neonates born to dams with prolonged Se deficient exhibit decreased pulmonary, renal, and cardiac GPx activity. C57Bl/6 mice were placed on diets that differed only in Se content, either 0.4 ppm or <0.01 ppm of sodium selenite. Breeding was initiated after 2–4 weeks on diet and natural delivery was allowed. Hepatic, cardiac, renal, and pulmonary organ homogenate was evaluated on day of birth. Each data point represents either a female or male from each litter. (**A**) Glutathione peroxidase (GPx) activity level by oxidation of NADPH per minute per milligram of protein in Se sufficient liver, heart, kidney, and lungs. (**B**) Cardiac glutathione peroxide activity level by oxidation of NADPH per minute per milligram of protein. (**C**) Renal glutathione peroxide activity level by oxidation of NADPH per minute per milligram of protein. (**D**) Pulmonary glutathione peroxide activity level by oxidation of NADPH per minute per milligram of protein N = 4 for all groups. Data and presented as mean (±SEM), * *p* < 0.05 vs. SeS control, by multiple comparison after one-way ANOVA, ∨ *p* < 0.05 vs. SeD <10 weeks by multiple comparisons after one-way ANOVA.

Adult models of Se deficiency demonstrate the presence of decreased selenoprotein expression in the heart, kidney, and lung in response to limited dietary Se supply. Given that GPx is exquisitely sensitive to Se bioavailability, we interrogated the impact of antenatal Se deficiency on GPx activity in the heart, kidney, and lung [24,46]. First, we compared relative GPx activity in livers, hearts, kidneys, and lungs from pups born to SeS dams (Figure 7A). GPx activity was greatest in the liver and heart at P0. Specifically, GPx activity in kidney and lung was, respectively, 30% and 38% lower than in the neonatal liver. Next, we measured GPx activity in these organs for pups born to dams after short or prolonged SeD. Cardiac and renal GPx activity appeared to be lower in pups born short maternal SeD when compared to SeS; however, the differences were not statistically significant (*p* = 0.08 for the heart and *p* = 0.1 for the kidney). Both cardiac and renal GPx activity were significantly lower in pups born to dams with prolonged SeD (Figure 7B,C). Pulmonary GPx activity was decreased in pups after both short and prolonged maternal SeD and was lowest in pups born after prolonged perinatal SeD. (Figure 7D).

## 4. Discussion

Maternal Se deficiency is associated with decreased neonatal Se status, which increases the risk for multiple neonatal morbidities [11,18,52,53]. This suggests an important role for Se during embryonic and neonatal development. Few studies have specifically interrogated the impact of isolated maternal Se deficiency on Se-dependent and Se-independent neonatal antioxidant defenses. Therefore, to determine the impact of maternal Se deficiency on neonatal selenoprotein expression, we investigated the impact of antenatal Se deficiency on neonatal circulating and organ specific selenoproteins, focusing on the liver. First, we demonstrated that selenoproteins in the neonatal circulation and liver were decreased by maternal Se deficiency. Neonatal circulating and hepatic GPx activity, and hepatic Sps2 and GPx1 protein contents were decreased in pups born to SeD dams. Second, we observed that neonatal hepatic Txnrd1 expression was variably impacted based on duration of maternal SeD diet. Though Txnrd1 expression was initially preserved, it was significantly decreased in pups born to dams exposed to a SeD diet for >10 weeks. Hepatic Non-Se containing antioxidant enzymes, including SOD activity and HO-1 content and activity are increased in SeD adult mice. Therefore, we next evaluated the impact of liver SeD on non-Se antioxidant enzymes (AOE) in pups from to SeD dams. We found that similar to the pattern observed for neonatal hepatic Txnrd1, the expression of other AOEs was unaltered by a short maternal duration SeD diet. In contrast, SOD-2 and HO-1 contents were increased in pups born to dams exposed to a longer duration SeD diet. Finally, we found that neonatal cardiac, renal, and pulmonary GPx activity were also varied based on duration of maternal SeD. These findings demonstrate neonatal pups born to Se deficient mothers are at risk for depletion in multiple Se- containing antioxidant enzymes. Additionally, neonates exhibit a unique selenoprotein expression pattern following maternal Se deficiency. Finally, these findings suggest that the duration or degree of maternal Se deficiency may exert a cumulative impact on the developing infant.

These experiments are the first to establish a neonatal hepatic selenoprotein hierarchy after exposure to decreased maternal Se supply. The two neonatal hepatic selenoproteins with the most significant decrease, thus identified as low in the neonatal Se hierarchy, were hepatic Sps2 and glutathione peroxidase 1 GPx1, as well as total GPx activity in the liver and circulation. Post-transcriptional modifications in the Sec synthesis machinery can limit the Sec content of other selenoproteins [54,55,56]. We speculate that the decrease in Sps2 may reflect an autoregulatory process or a negative feedback mechanism during neonatal states of low Se availability. Our findings for GPx are consistent with adult studies, wherein the GPx family is highly sensitive to alterations in Se status, and Se deficiency induces a transcriptionally regulated decrease in Gpx1 content and activity [48,51]. This speculation is also supported by a report that maternal Se deficiency throughout both pregnancy and lactation decreased hepatic GPx activity in weanling rats [57]. GPx1 knock-out (KO) mice are fertile and produce neonatal mice that appear phenotypically normal [58]. Cumulatively, the existing literature as well as evidence from our study indicate that hepatic GPx1 is not indispensable in the developing mouse [58]. Studies in adult rodents demonstrate that GPx1 deficiency exacerbates injury induced by ischemia/reperfusion, diquat and paraquat exposure, and viral myocarditis [59,60,61,62]. Thus, we speculate that decreased neonatal circulating and hepatic GPx may become maladaptive if the infant were clinically exposed to high oxidant stress, as is often the case in modern neonatal intensive care units.

In contrast to neonatal hepatic Sps2 and GPx1, hepatic Txnrds were less sensitive to maternal Se deficiency. Txnrd1 protein content was sustained in pups born to SeD dams during their first or second gestation and Txnrd2 protein content was unaltered in both short and prolonged Se deficiency. The preservation of hepatic Txnrd2 is consistent with previous reports in adult Se deficiency models [63]. The response for Txnrd1 differs both in comparison to adult SeD rodents, as well as to our data in breeding aged mice, which demonstrated a decline in Txnrd1 content after only 2–4 weeks of Se deficiency [23,34]. This discrepancy in Txnrd1 may indicate a teleologic adaption to maintain neonatal hepatic Txnrd1 content during early maternal Se deficiency. While it is not currently known if either hepatic-specific Trxrd 1 or 2 are essential for embryogenesis, germline deletion of Txnrd1 or Txnrd2 is fatal in utero, and Txnrd2 KO mice demonstrate hepatic apoptosis, supporting their importance during early development [64,65]. Other models demonstrate the Txnrds are preferentially preserved in settings of low Se supply [25].

We observed a variable response to the duration of maternal Se deficiency for hepatic Txnrd1 contents as well as GPx activities in the heart, kidney, and lung. Neonatal hepatic Txnrd1 contents eventually declined in pups born to dams maintained on a SeD diet >10 weeks. Similarly, cardiac and renal GPx activities were not significantly lower in pups born to dams with short SeD exposure but were decreased in pups born to dams with prolonged SeD exposure. GPx activity in the neonatal lung was lower in pups born to dams after short maternal SeD duration and this decrease was exacerbated by prolonged maternal SeD diet. One possible explanation for these observations is that the duration of SeD dictates the severity of maternal Se deficiency, resulting in a greater competition for available Se atoms between mother and infant. This explanation is supported by past work demonstrating progressive impact of the duration of Se deficiency [27,46]. Alternatively, our observations may reflect a confounding factor of maternal age, and the possibility that younger SeD dams may accommodate more efficient placental Se transfer than do older SeD dams. Future studies will clarify specific neonatal responses to the duration of maternal SeD. However, we speculate that inadequate maternal Se status may become progressively detrimental to the neonate, depending on duration and degree of Se deficiency.

When one or more AOE are decreased, adaptive compensation can occur [66,67]. Thus, we tested the effect of neonatal SeD on hepatic expression of other AOE important in hydrogen peroxidase regulation or known to increase in the adult SeD liver. Our observations demonstrated a divergent response in alternative AOEs that varied depending on duration of maternal SeD exposure. Pups born to dams receiving SeD diet for <10 weeks demonstrated unaltered hepatic protein content of SODs, catalase, and HO-1. However, pups born to dams exposed to SeD for >10 weeks demonstrated an increase in hepatic SOD2 and HO-1 contents. The neonatal hepatic response to prolonged maternal SeD is similar to the adult SeD liver, in which SOD activity as well as HO-1 protein content and activity are increased [24,38]. There are several possible explanations for why the neonatal SeD liver responds in a divergent manner based on duration of maternal exposure. Despite the striking decrease in neonatal hepatic GPx content and activity, there may not be a sufficient enought increase in oxidative stress to induce SOD2 and HO-1. While others have reported that decreased Se status and GPx activity is associated with increased markers of oxidative stress in adults, these studies have not tested the impact of isolated GPx deficiency on redox state [29,50,51]. It is also possible that decreased Txnrd1 contents after Se deficiency drives Nrf2-dependent compensatory responses. Prior in vivo and in vitro work with genetic and pharmacologic Txnrd1 inhibition demonstrate that decreased hepatic Txnrd1 induces HO-1 [68]. Our future work will further elucidate the impact of Se deficiency on the hepatic redox state under normal physiologic conditions and in settings of oxidative stress.

The biologic impact of sex is increasingly recognized as a key determinant of human health and disease. Sex-specific dimorphisms for factors influencing Se processing, recycling, and selenoprotein expression have been reported in adult rodents [25]. Our results are consistent with prior reports that adult females demonstrate increased hepatic GPx activity at baseline. Additionally, we demonstrated that female SeD breeding age dams exhibited higher hepatic GPx activity than male SeD breeding age mice. In contrast to these results, we found no sex-dependent differences in the transcription, protein content, or activity of GPx in neonatal mice. Additionally, our data indicated no sex-dependent effects on protein contents of Txnrd1, Txnrd2, or on factors for Sec processing in neonatal mice, under conditions of Se sufficiency or deficiency. Currently, it is not known specifically at what age sex differences emerge for hepatic GPx activity. Additionally, the molecular mechanism for Se-related sex differences in adult studies remains unclear. Evaluation of rodents after gonadectomy demonstrates variable results, but some prior work supports a component of estrogenic and androgenic regulation [47,69]. In vitro work in hepatocytes indicated the possible contribution of steroid-independent mechanisms [70]. Regardless, our data imply that the mechanisms responsible for the adult sex differences in GPx do not appear to be present in the neonatal period. However, these differences are present as early as 6–8 weeks of life, as demonstrated in breeding aged mice. Meticulous evaluation for sex differences in relation to Se processing, transport, and selenoprotein expression after development will be important for future work.

Cumulatively, our findings demonstrate that neonatal pups born to Se deficient dams are born with diminished selenoenzyme defenses. We speculate this may have important clinical implications. Maternal Se deficiency can occur with maternal malnutrition or secondary to antenatal states such as pre-eclampsia and gestational diabetes [39,41,52,71]. Furthermore, the majority of fetal Se acquisition occurs during the third trimester, thus infants born premature are at high risk for Se deficiency [41]. Our findings demonstrate that insufficient Se supply in the perinatal period results in decreased circulating GPx as well as hepatic GPx and eventually Trxrd1. As infants can experience a myriad of oxidative challenges, insufficient selenoenzyme defense may increase neonatal morbidities or mortality [17]. Neonatal Se levels are not routinely measured as standard of care [41]. Whether circulating GPx could be used as a biomarker to identify infants with Se deficiency is an important area for future investigation. As Se can be supplemented both enterally and parenterally, this may represent a modifiable nutritional intervention.

We focused this study on several selenoproteins recognized as important in the adult liver and defined a unique response in the neonate within this targeted evaluation. Our study is limited by an incomplete evaluation of neonatal Se hierarchy as we did not examine the levels of every hepatic selenoprotein. Another limitation is that Txnrd contents and activity do not always correlate. While future work will need to evaluate if the preserved Txnrd1 content correlates with sustained activity, it is interesting that a short duration of maternal SeD did not alter either Txnrd1 content or other antioxidant enzymes, whereas prolonged exposure resulted in divergent responses in both. As our findings indicate that the cumulative duration of maternal SeD is an important variable for the degree of selenoprotein depletion in the neonate, this will need to be tightly controlled for in future experiments. Finally, we did not perform Se isotype tracer studies to evaluate protein half-life, turnover, and de novo selenoprotein synthesis in the neonate, nor have we sequentially evaluated the degree of Se deficiency in the dams undergoing serial gestations. Both would be interesting directions for future work to add to the growing understanding of periconception and antenatal maternal Se status and the impact on the neonate.

## 5. Conclusions

We conclude that offspring born after maternal periconception and antenatal Se deficiency exhibit decreased circulating and organ-specific selenoproteins. Neonatal circulating and hepatic GPx1 are highly sensitive to maternal Se deficiency, which may render infants at risk for increased morbidity after oxidative challenge. Neonatal mice demonstrate a different selenoprotein expression pattern compared to adults, with initial preservation in hepatic Txnrd1 content. The impact on the offspring varies depending on the duration of maternal exposure to the deficient diet. This study indicates that the duration and degree of maternal Se deficiency may exert a progressive impact on the developing infant.

## Data Availability

The data is available from the author by request.

## Supplementary Materials

The following are available online at https://www.mdpi.com/2076-3921/10/2/288/s1, Supplemental Table S1: Primers for qPCR, Real-Time PCR details, and Primary Antibodies for Western blots; Figure S1: Circulating GPx activity after antenatal Se deficiency, analyzed by sex; Figure S2: Neonatal hepatic transcription for factors for selenocysteine synthesis after antenatal Se deficiency, analyzed by sex; Figure S3: Neonatal hepatic glutathione peroxidase 1 is decreased by antenatal Se deficiency, analyzed by sex; Figure S4: Neonatal hepatic thioredoxin reductases after antenatal Se deficiency, analyzed by sex; Figure S5: Neonates born to dams with prolonged Se deficient exhibit increased hepatic SOD2 and HO-1, analyzed by sex; Figure S6: Circulating GPx activity after antenatal Se deficiency, data with 95% confidence interval. Figure S7: Neonatal hepatic transcription for factors for selenocysteine synthesis after antenatal Se deficiency, data with 95% confidence interval; Figure S8: Neonatal hepatic glutathione peroxidase 1 is decreased by antenatal Se deficiency, data with 95% confidence intervals; Figure S9: Neonatal hepatic thioredoxin reductases after antenatal Se deficiency, with 95% confidence intervals; Figure S10: Neonates born to dams with prolonged Se deficient exhibit increased hepatic SOD2 and HO-1, data with 95% confidence intervals.
