# Supplementary material for: Neonatal Selenoenzyme Expression Is Variably Susceptible to Duration of Maternal Selenium Deficiency
Source: Antioxidants (Basel). 2021 Feb 14;10(2):288. doi: 10.3390/antiox10020288 (PMC7917816; doi:10.3390/antiox10020288)
Supplement: Supplementary file 1 [file antioxidants-10-00288-s001.pdf]

**Taqman Primers**	
Gpx1	Mm00656767_m1
Pstk	Mm00617112_m1
Scly	Mm00489563_m1
Selenop	Mm00486048_m1
SepsecS	Mm00552788_m1
Sephs2	Mm00545980_s1
TrxR1	Mm00443675_m1
TrxR2	Mm00496766_m1

**Real- Time PCR**		
Temperature	Time	Cycles
50 degrees Celsius	2 minutes	1
95 degrees Celsius	2 minutes	1
95 degrees Celsius	1 second	40
60 degrees Celsius	20 seconds	

**Primary Antibodies for Western blots**		
Glutathione peroxidase 1	1:500	R&D Systems, Minneapolis, MN
Pstk	1:500	LSBio, Seattle, WA
Selenocysteine Lyase	1:500	Santa Cruz, Santa Cruz, CA
Selenoprotein P	1:1000	generous gift from Dr. Yoshiro Saito and Dr. Hiraoki
SepsecS	1:1000	LSBio, Seattle, WA
Selenophosphate Synthetase 2	1:1000	1:1000, Rockland Institute, Limerick, PA
Thioredoxin reductase 1	1:2000	TE Tipple lab, Oklahoma City, OK
Thioredoxin reductase 2	1:500	Santa Cruz, Santa Cruz, CA
SOD1	1:1000	Abcam, Cambridge, UK
SOD2	1:500	Millipore, Billerica, MA
SOD3	1:500	R&D Systems, Minneapolis, MN
catalase	1:1000	Abcam, Cambridge, UK
HO-1	1:1000	Cell Signaling, Danvers, MA

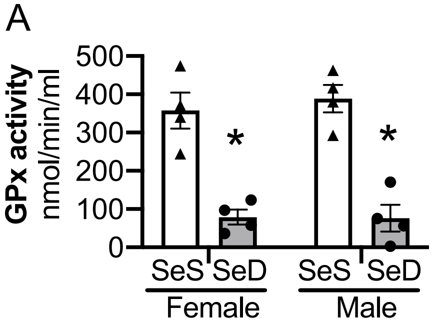

Supplemental figure 1: Circulating GPx activity after antenatal Se deficiency, analyzed by sex. C57Bl/6 mice were placed on diets that differed only in Se content, either 0.4ppm or <0.01 ppm of sodium selenite. Breeding was initiated after 2-4 weeks on diet and natural delivery was allowed. (**A)** Plasma glutathione peroxidase activity level by oxidation of NADPH per minute. Each data point represents either a female or male from each litter; each individual point is an average of two mice N = 4-5 for all groups. Data and presented as mean (±SEM), * $p<0.05$ vs. sex-matched SeS control, by multiple comparisons after two-way ANOVA.

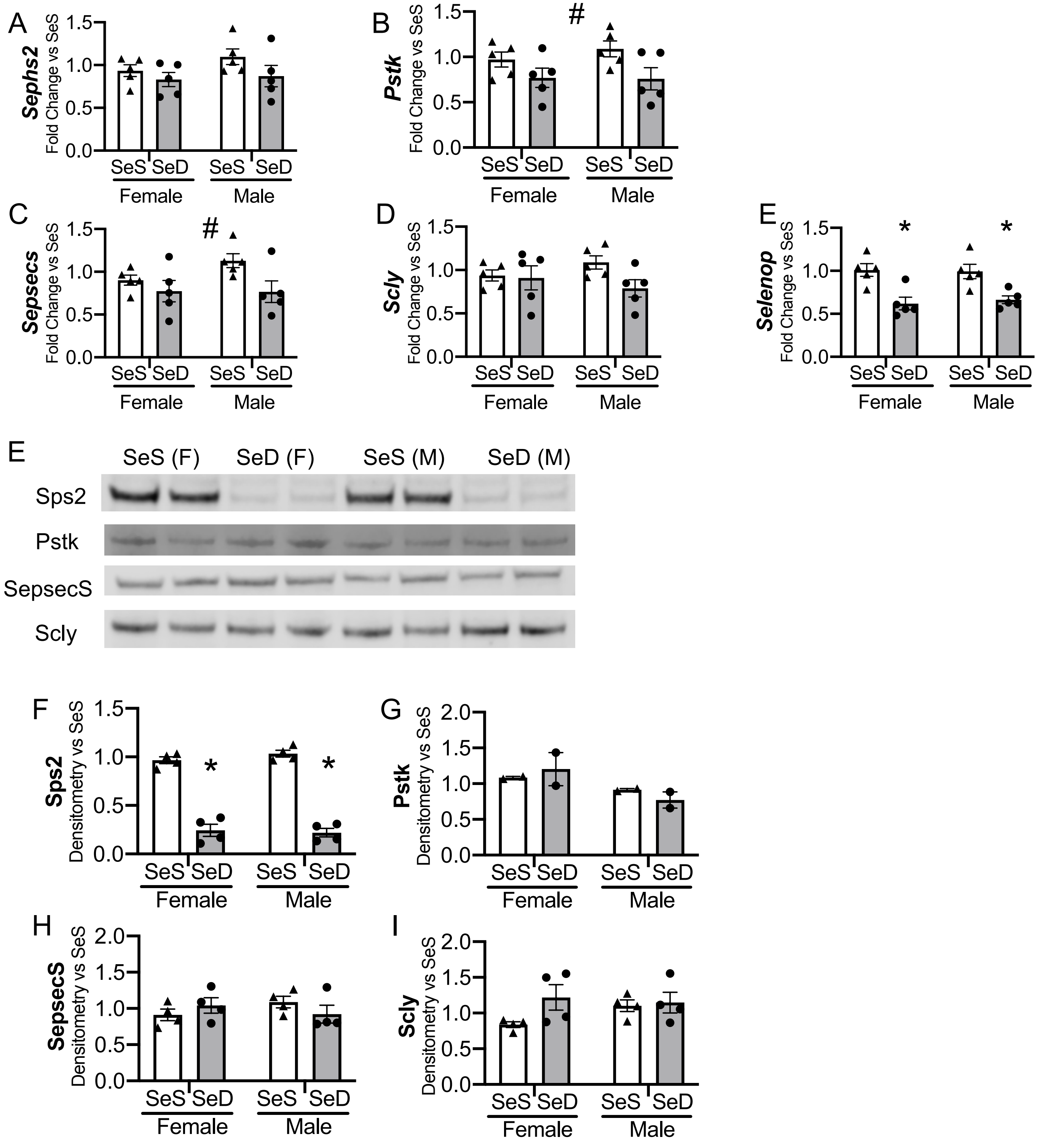

Supplemental figure 2: Neonatal hepatic transcription for factors for selenocysteine synthesis after antenatal Se deficiency, analyzed by sex. C57Bl/6 mice were placed on diets that differed only in Se content, either 0.4ppm or <0.01 ppm of sodium selenite. Breeding was initiated after 2-4 weeks on diet and natural delivery was allowed. Hepatic organ homogenate was evaluated on day of birth. Each data point represents either a female or male from each litter; each individual point is an average of two mice. Fold change in (**A**) *Sephs2*, **(B)** *Pstk,* **(C)** *Sepsecs* mRNA, (**D**) *Scly* mRNA and **(E)** *Selenop* mRNA are shown normalized to SeS samples. **(E)** Representative Western blots of hepatic Sps2, Pstk, SepsecS and Scly. Densitometric analysis of **(F)** Sps2, **(G)** Pstk, **(H)** SepsecS and **(I)** Scly protein content expression. Results are normalized to total protein stain and expressed as a ratio to SeS mice. N = 2-5 for all groups Data and presented as mean (±SEM), # p <0.05 by two-way ANOVA for diet, * p<0.05 vs. sex-matched SeS control, by multiple comparisons after two-way ANOVA.

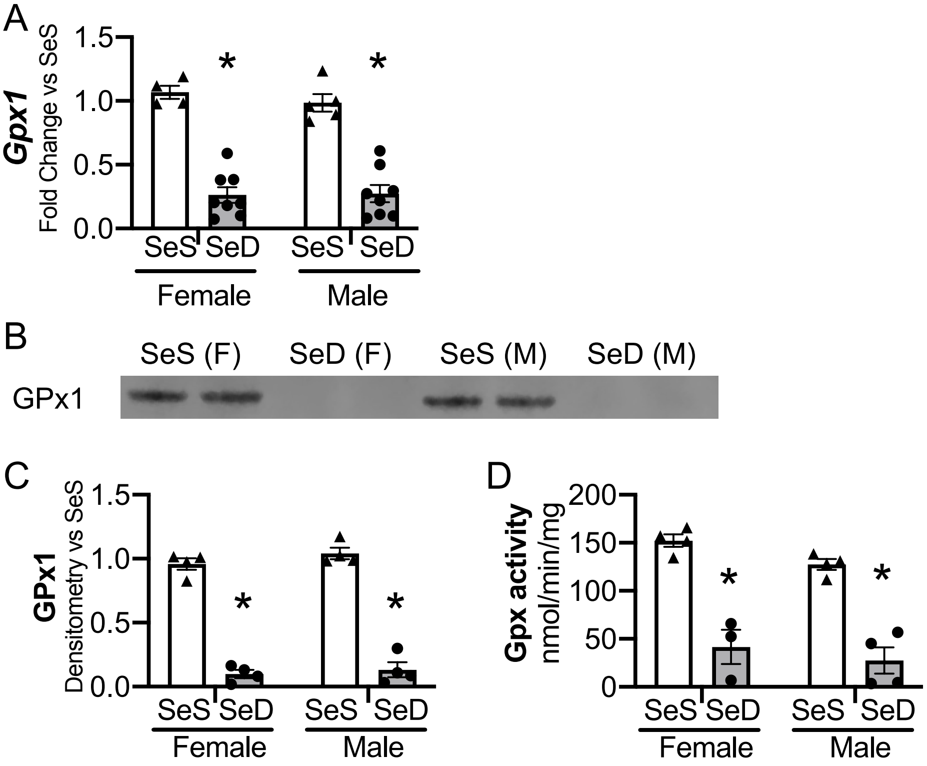

Supplemental figure 3. Neonatal hepatic glutathione peroxidase 1 is decreased by antenatal Se deficiency, analyzed by sex. C57Bl/6 mice were placed on diets that differed only in Se content, either 0.4ppm or 0 ppm of sodium selenite. Breeding was initiated after 2-4 weeks on diet and natural delivery was allowed. Hepatic organ homogenate was evaluated on day of birth. Each data point represents the average results for two males or 2 females from each litter. **(A)** Fold change in *Gpx1* mRNA expression is shown normalized to SeS samples. (**B)** Representative Western blot of hepatic Gpx1 Densitometric analysis of **(C)** Gpx1 protein content expression. Results are normalized to total protein stain and expressed as a ratio to Se sufficient mice. **(D)** Glutathione peroxide activity level by oxidation of NADPH per minute per milligram of protein. N = 4-8 for all groups. Data and presented as mean (±SEM), * $p<0.05$ vs. sex-matched SeS control, by multiple comparisons after two-way ANOVA.

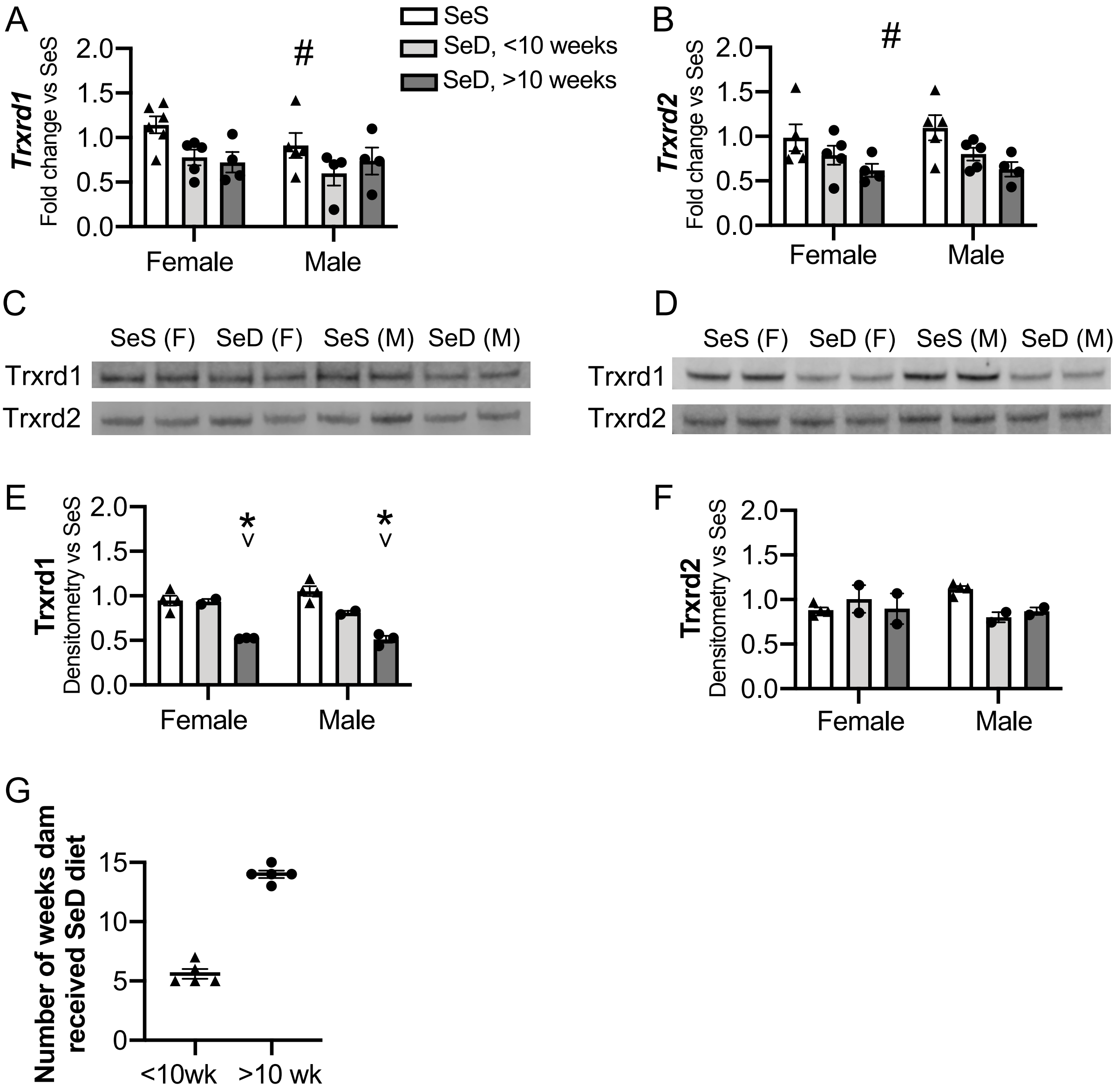

Supplemental figure 4. Neonatal hepatic thioredoxin reductases after antenatal Se deficiency, analyzed by sex. C57Bl/6 mice were placed on diets that differed only in Se content, either 0.4ppm or <0.01 ppm of sodium selenite. Breeding was initiated after 2-4 weeks on diet and natural delivery was allowed. Hepatic organ homogenate was evaluated on day of birth. Analysis done with SeD samples separated based on if the breeding dam received more or less than 10 weeks SeD diet at time of birth. Each data point represents either a female or male from each litter; each individual point is an average of two mice. **(A)** Fold change in *Trxrd1* mRNA and **(B)** *Trxrd2* mRNA expression is shown normalized to SeS samples, (**C**) Representative Western blot of hepatic Trxrd1 and Trxrd2 for SeS samples and SeD samples born to dams who received <10 weeks SeD diet, (**D**) Representative Western blot of hepatic Trxrd1 and Trxrd2 for SeS samples and SeD samples born to dams who received >10 weeks SeD diet, Densitometric analysis of **(E)** Trxrd1 and **(F)** Trxrd2 protein content expression. Results are normalized to total protein stain and expressed as a ratio to Se sufficient mice. **(G)** Duration of SeD exposure for dam, short versus long SeD groups. N = 2-6 for all groups. Data and presented as mean (±SEM), # p <0.05 by two-way ANOVA for diet, * p<0.05 vs SeS control by multiple comparisons after two-way ANOVA, $_{v}$ p <0.05 vs SeD <10 weeks by multiple comparisons after two-way ANOVA

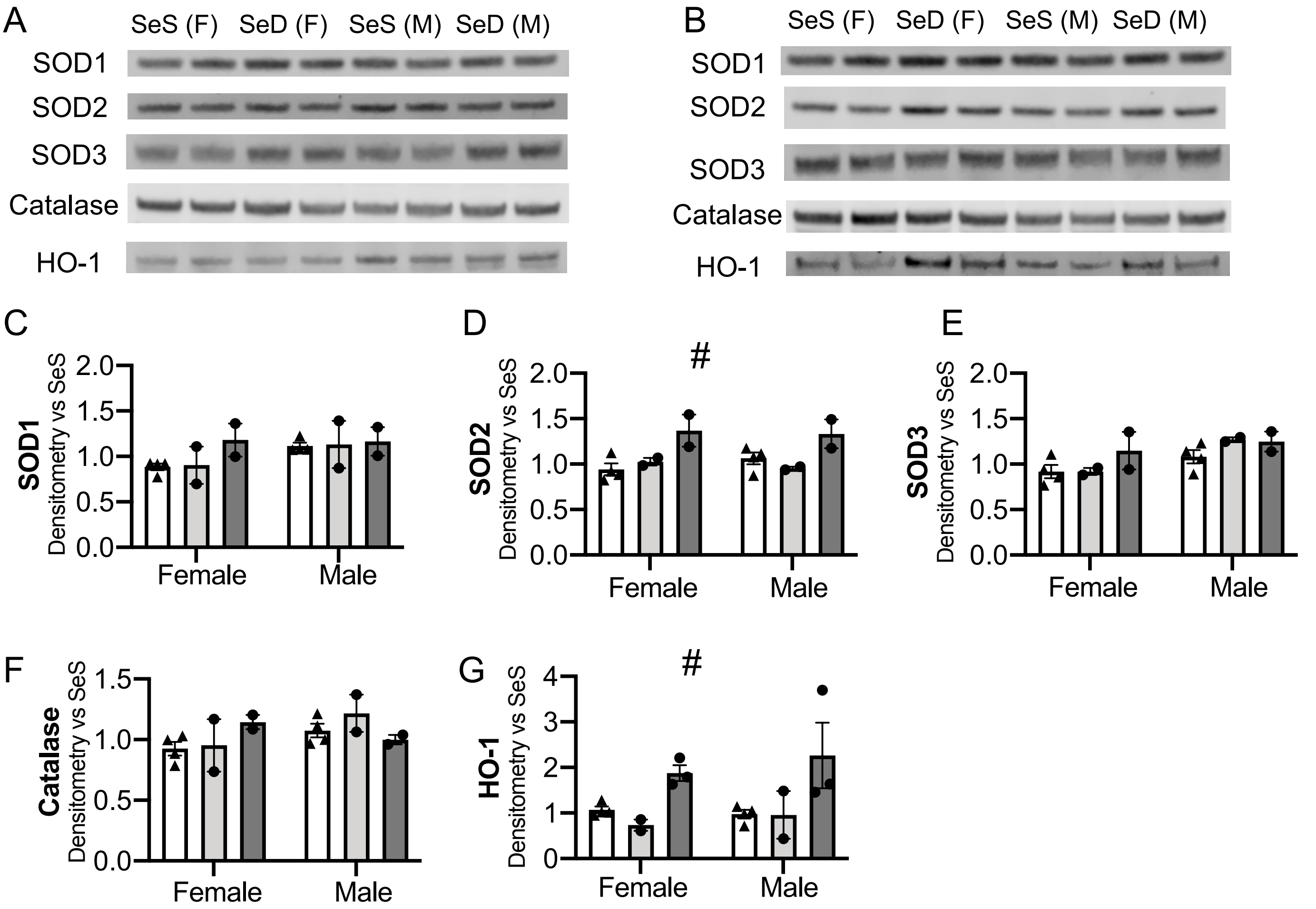

Supplemental figure 5: Neonates born to dams with prolonged Se deficient exhibit increased hepatic SOD2 and HO-1, analyzed by sex. C57Bl/6 mice were placed on diets that differed only in Se content, either 0.4ppm or <0.01 ppm of sodium selenite. Breeding was initiated after 2-4 weeks on diet and natural delivery was allowed. Hepatic organ homogenate was evaluated on day of birth. Each data point represents either a female or male from each litter; each individual point is an average of two mice. (**A**) Representative Western blots of hepatic SOD1, SOD2, SOD3, catalase, HO-1 for SeS pups and pups born to dams receiving <10 weeks SeD diet. (**B**) Representative Western blots of hepatic SOD1, SOD2, SOD3, catalase, HO-1 for SeS pups and pups born to dams receiving >10 weeks SeD diet. Densitometric analysis of **(C)** SOD1, **(D)** SOD2, **(E)** SOD3, and **(F)** Catalase, **(G)** HO-1 protein content expression. Results are normalized to total protein stain and expressed as a ratio to SeS mice, N= 2-4 for all groups. Data and presented as mean (±SEM), # p <0.05 by two-way ANOVA for diet

A

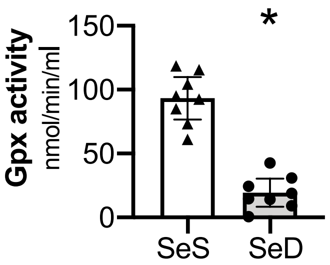

Supplemental figure 6: Circulating GPx activity after antenatal Se deficiency, data with 95% confidence interval. C57Bl/6 mice were placed on diets that differed only in Se content, either 0.4ppm or <0.01 ppm of sodium selenite. Breeding was initiated after 2-4 weeks on diet and natural delivery was allowed. (**A)** Plasma glutathione peroxidase activity level by oxidation of NADPH per minute. Each data point represents either a female or male from each litter; each individual point is an average of two mice N = 6-8 for all groups. Data and presented as mean (±95% confidence interval), * $p<0.05$ vs. SeS control, by two-sided t test.

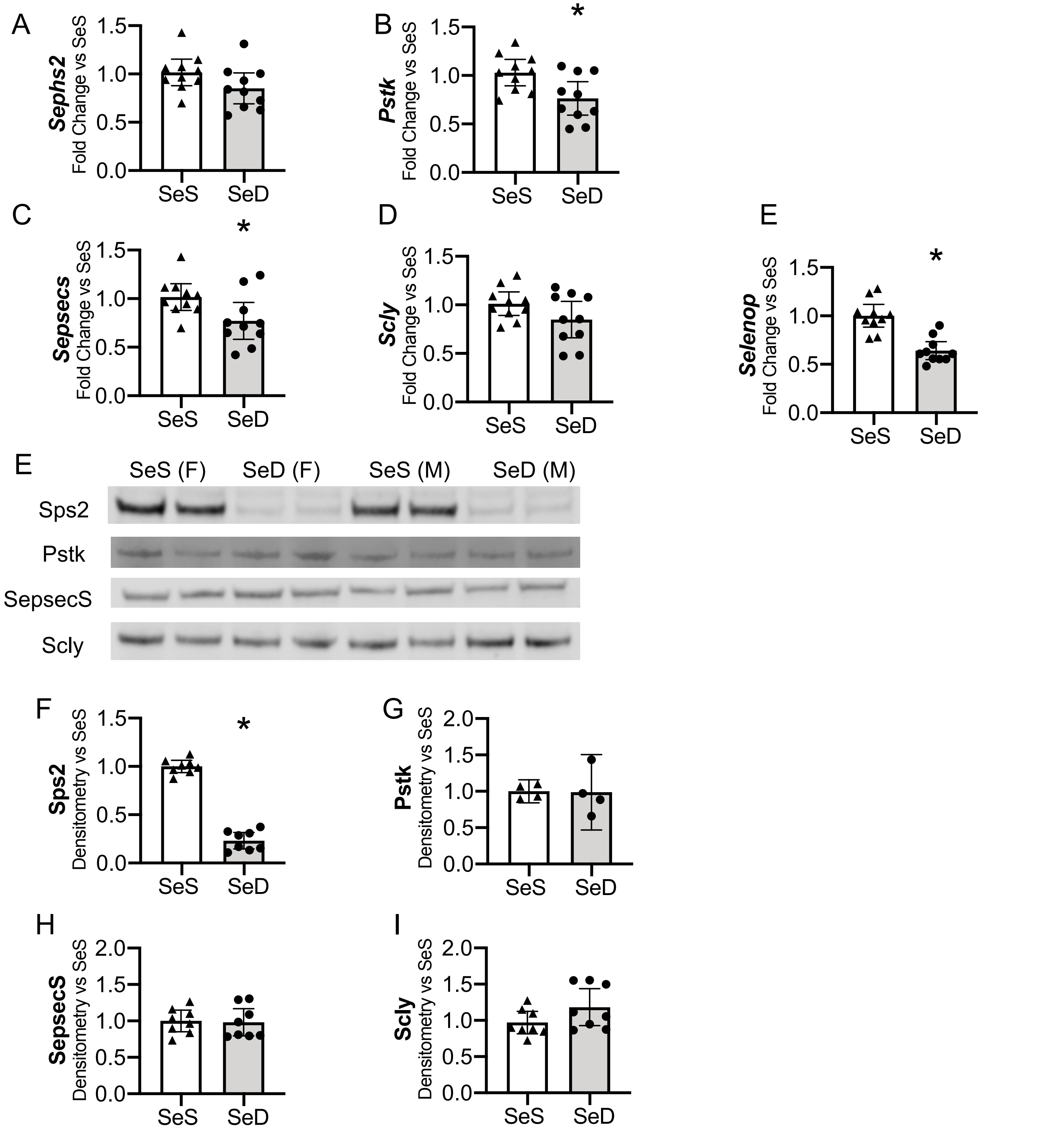

Supplemental figure 7: Neonatal hepatic transcription for factors for selenocysteine synthesis after antenatal Se deficiency, data with 95% confidence interval. C57Bl/6 mice were placed on diets that differed only in Se content, either 0.4ppm or <0.01 ppm of sodium selenite. Breeding was initiated after 2-4 weeks on diet and natural delivery was allowed. Hepatic organ homogenate was evaluated on day of birth. Each data point represents either a female or male from each litter; each individual point is an average of two mice. Fold change in (**A)** *Sephs2*, **(B)** *Pstk,* **(C)** *Sepsecs* mRNA, (**D)** *Scly* mRNA and **(E)** *Selenop* mRNA are shown normalized to SeS samples. **(E)** Representative Western blots of hepatic Sps2, Pstk, SepsecS and Scly. Densitometric analysis of **(F)** Sps2, **(G)** Pstk, **(H)** SepsecS and **(I)** Scly protein content expression. Results are normalized to total protein stain and expressed as a ratio to SeS mice. N = 4-10 for all groups. Data and presented as mean (±95% confidence interval), * p<0.05 vs. SeS control, by two-sided t test.

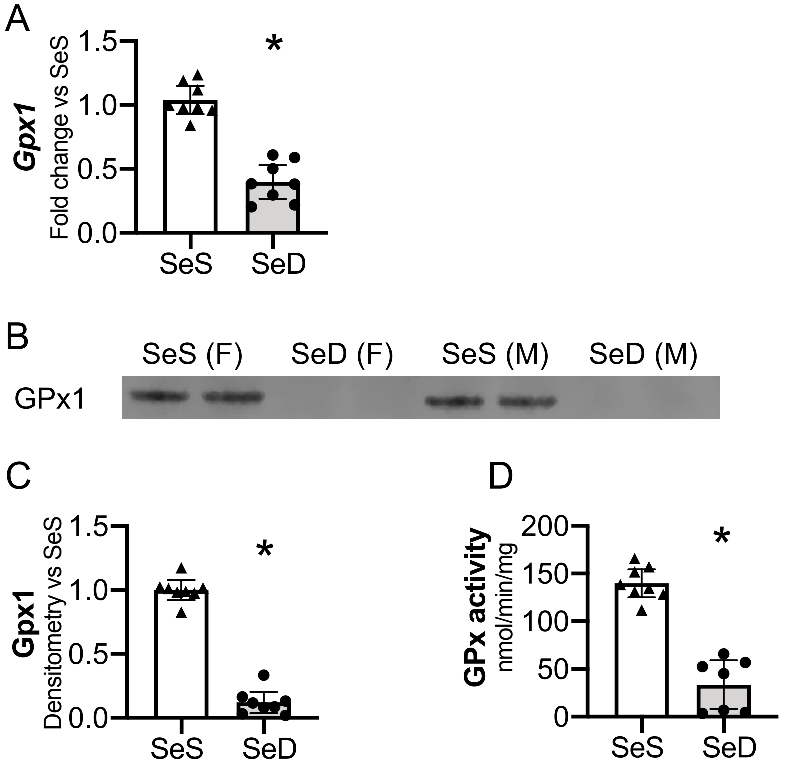

Supplemental figure 8. Neonatal hepatic glutathione peroxidase 1 is decreased by antenatal Se deficiency, data with 95% confidence intervals. C57Bl/6 mice were placed on diets that differed only in Se content, either 0.4ppm or 0 ppm of sodium selenite. Breeding was initiated after 2-4 weeks on diet and natural delivery was allowed. Hepatic organ homogenate was evaluated on day of birth. Each data point represents the average results for two males or 2 females from each litter. **(A)** Fold change in *Gpx1* mRNA expression is shown normalized to SeS samples. (**B)** Representative Western blot of hepatic Gpx1 Densitometric analysis of **(C)** Gpx1 protein content expression. Results are normalized to total protein stain and expressed as a ratio to Se sufficient mice. **(D)** Glutathione peroxide activity level by oxidation of NADPH per minute per milligram of protein. N = 7-8 for all groups. Data and presented as mean (±95% confidence interval), * p<0.05 vs. SeS control, by two-sided t test.

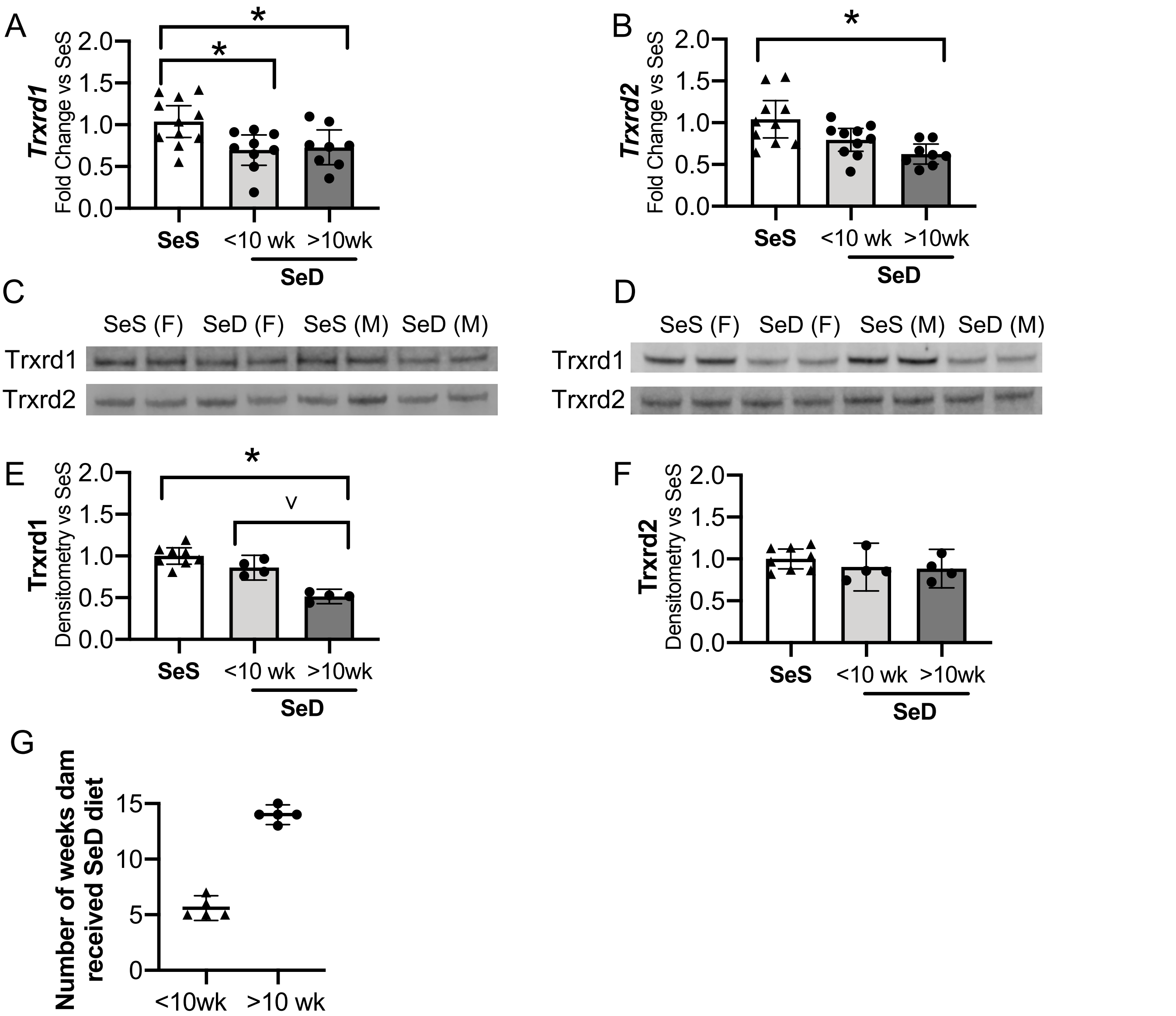

Supplemental figure 9. Neonatal hepatic thioredoxin reductases after antenatal Se deficiency, with 95% confidence intervals. C57Bl/6 mice were placed on diets that differed only in Se content, either 0.4ppm or <0.01 ppm of sodium selenite. Breeding was initiated after 2-4 weeks on diet and natural delivery was allowed. Hepatic organ homogenate was evaluated on day of birth. Analysis done with SeD samples separated based on if the breeding dam received more or less than 10 weeks SeD diet at time of birth. Each data point represents either a female or male from each litter; each individual point is an average of two mice. **(A)** Fold change in *Trxrd1* mRNA and **(B)** *Trxrd2* mRNA expression is shown normalized to SeS samples, (**C)** Representative Western blot of hepatic Trxrd1 and Trxrd2 for SeS samples and SeD samples born to dams who received <10 weeks SeD diet, **(D)** Representative Western blot of hepatic Trxrd1 and Trxrd2 for SeS samples and SeD samples born to dams who received >10 weeks SeD diet, Densitometric analysis of **(E)** Trxrd1 and **(F)** Trxrd2 protein content expression. **(G)** Duration of SeD exposure for dam, short versus long SeD groups. Results are normalized to total protein stain and expressed as a ratio to Se sufficient mice. N = 4-10 for all groups. Data and presented as mean (±95% confidence interval), * $p<0.05$ vs SeS control by multiple comparisons after one-way ANOVA, $_v$ $p <0.05$ vs SeD <10 weeks by multiple comparisons after one-way ANOVA

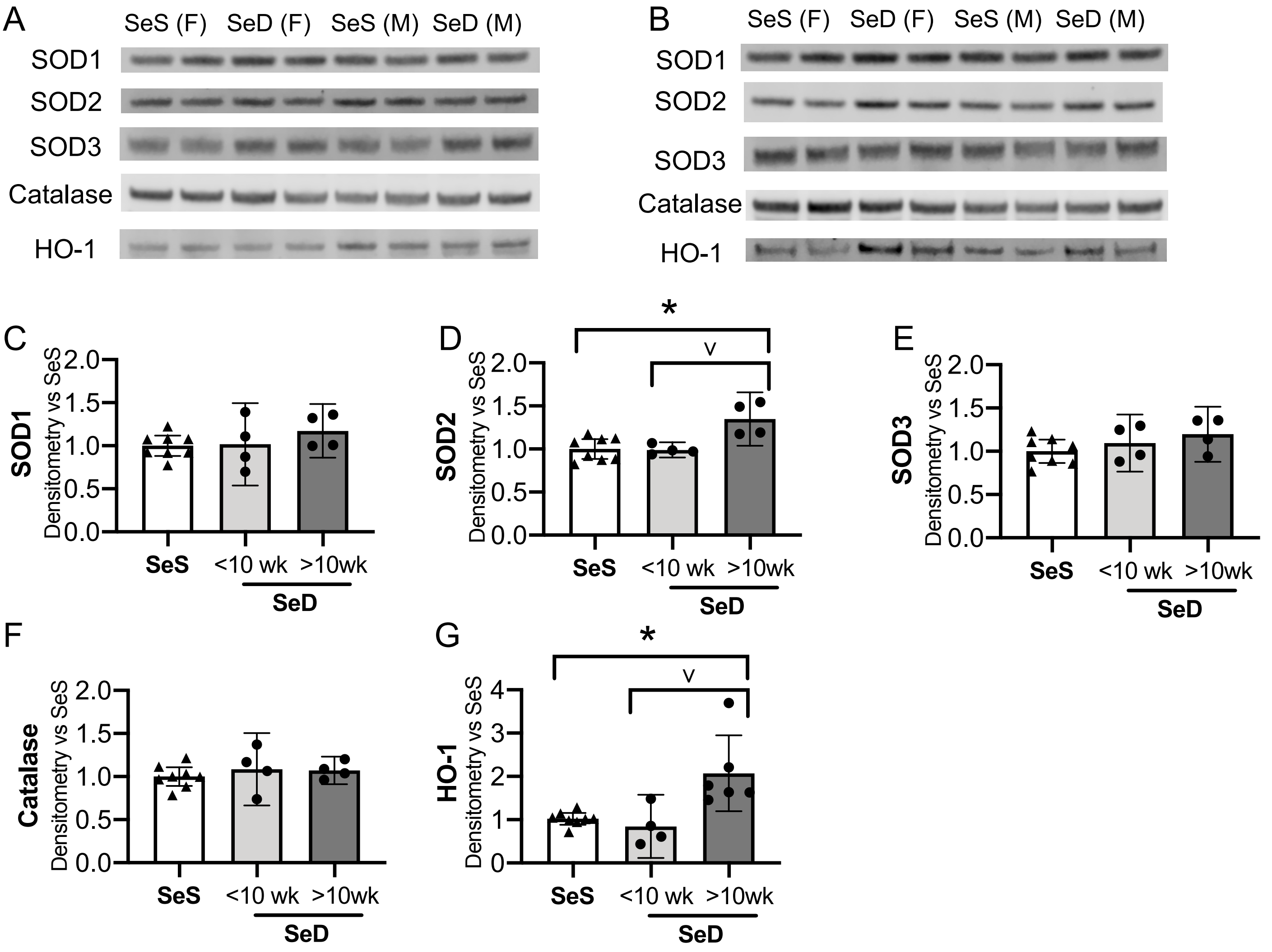

Supplemental figure 10: Neonates born to dams with prolonged Se deficient exhibit increased hepatic SOD2 and HO-1, data with 95% confidence intervals. C57Bl/6 mice were placed on diets that differed only in Se content, either 0.4ppm or <0.01 ppm of sodium selenite. Breeding was initiated after 2-4 weeks on diet and natural delivery was allowed. Hepatic organ homogenate was evaluated on day of birth. Each data point represents either a female or male from each litter; each individual point is an average of two mice. (**A**) Representative Western blots of hepatic SOD1, SOD2, SOD3, catalase, HO-1 for SeS pups and pups born to dams receiving <10 weeks SeD diet. (**B**) Representative Western blots of hepatic SOD1, SOD2, SOD3, catalase, HO-1 for SeS pups and pups born to dams receiving >10 weeks SeD diet. Densitometric analysis of **(C)** SOD1, **(D)** SOD2, **(E)** SOD3, and **(F)** Catalase, **(G)** HO-1 protein content expression. Results are normalized to total protein stain and expressed as a ratio to SeS mice, N= 4-8 for all groups. Data and presented as mean (±95% confidence interval), * $p<0.05$ vs SeS control by multiple comparisons after one-way ANOVA, $_{v}$ $p <0.05$ vs SeD <10 weeks by multiple comparisons after one-way ANOVA
